# Identity prediction errors in the human midbrain update reward-identity expectations in the orbitofrontal cortex

**DOI:** 10.1038/s41467-018-04055-5

**Published:** 2018-04-23

**Authors:** James D. Howard, Thorsten Kahnt

**Affiliations:** 1 Department of Neurology, Northwestern University, Feinberg School of Medicine, Chicago, IL 60611 USA; 2 Department of Psychology, Northwestern University, Weinberg College of Arts and Sciences, Evanston, IL 60208 USA

## Abstract

There is general consensus that dopaminergic midbrain neurons signal reward prediction errors, computed as the difference between expected and received reward value. However, recent work in rodents shows that these neurons also respond to errors related to inferred value and sensory features, indicating an expanded role for dopamine beyond learning cached values. Here we utilize a transreinforcer reversal learning task and functional magnetic resonance imaging (fMRI) to test whether prediction error signals in the human midbrain are evoked when the expected identity of an appetitive food odor reward is violated, while leaving value matched. We found that midbrain fMRI responses to identity and value errors are correlated, suggesting a common neural origin for these error signals. Moreover, changes in reward-identity expectations, encoded in the orbitofrontal cortex (OFC), are directly related to midbrain activity, demonstrating that identity-based error signals in the midbrain support the formation of outcome identity expectations in OFC.

## Abstract (web summary)

Responses in the dopaminergic midbrain are known to signal prediction errors for reward value. Here, the authors show that the human midbrain also encodes errors in predicted reward identity, and that these signals update expectations of reward identity in the orbitofrontal cortex.

## Introduction

Over the last two decades, activity of dopaminergic midbrain neurons has become almost synonymous with reward prediction errors^1,2^, signaling the difference between expected and received reward^3–5^. Such prediction errors are a key component of many reinforcement learning algorithms^6,7^, wherein they update associations between predictive events and rewarding outcomes.

These signals are typically described in terms of reward value prediction errors, restricting their use to learning associations between events and cached (model-free) value. A mechanism that only computes errors in cached value cannot account for the learning of complex associative structures that facilitate model-based behavior^8–12^. However, recent experiments in animals demonstrate that midbrain dopamine responds to errors in inferred value^13–15^ and is necessary for learning of value-neutral associations^16^. Most strikingly, dopamine neurons in rats have recently been shown to also respond in situations where value remains the same but the sensory features of the predicted reward change^17^. This suggests a much broader role for midbrain error signals beyond cached value to include the learning of a wide range of associations that together form a model of the task state.

One state feature that is particularly important for model-based behavior is the identity of future outcomes. For instance, information about outcome identity is necessary for adaptive behavior following devaluation, a hallmark of goal-directed behavior^18–21^. The orbitofrontal cortex (OFC) is critical for representing the identity of expected rewards along with other features of the task state^8,9,21–29^. However, the mechanisms by which task state features such as identity are learned and updated in the OFC remain unclear. Here we tested the hypothesis that the human midbrain responds to errors in predicted reward identity, and that these identity prediction errors are directly related to updating identity expectations in the OFC.

Hungry participants performed a transreinforcer reversal learning task during functional magnetic resonance imaging (fMRI) involving different types of value-matched food odors as rewards. Unexpectedly throughout the task, participants experienced violations either in expected reward identity while leaving value unaltered, or in expected reward value while leaving identity unchanged. Using this approach, we demonstrate that the human midbrain responds to value-neutral errors in identity predictions, and that these signals are correlated with changes in identity expectations in the OFC.

## Results

### Odor reward selection and experimental design

**Fig. 1:**
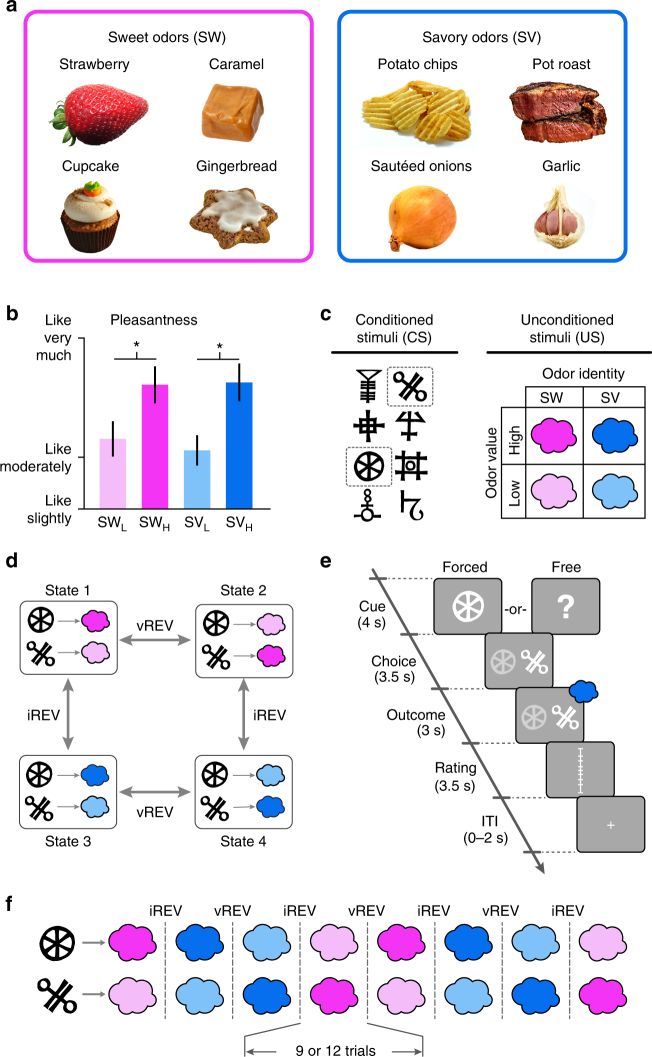
Odor selection and transreinforcer reversal learning task. **a** From an initial set of eight food odors, one sweet (SW) and one savory (SV) odor was chosen for each participant (*n* = 23) such that they were matched in rated pleasantness. A high-intensity and low-intensity version of each selected odor was then established. **b** High-intensity odors (SW_H_, SV_H_) were rated as more pleasant than low-intensity odors (SW_L_, SV_L_), and matched in pleasantness within intensity level. Error bars depict within-subject s.e.m. *p < 0.05 in post-hoc paired *t*-tests. **c** Two visual symbols (randomly selected for each participant) were used as conditioned stimuli (CS). The four food odors, comprising a 2 × 2 factorial design with value and identity as factors, were used as unconditioned stimuli (US). **d** Specific CS–US associations conformed to four unique states in the reversal learning task. Transitions between states followed either identity reversals (iREV) or value reversals (vREV). **e** On each trial, participants first saw a pre-choice “cue” to indicate whether it was a forced-choice or free-choice trial. They then chose one of the two CS’s to receive its deterministically paired odor US. **f** A given state persisted for either 9 or 12 trials, and states were separated by iREV or vREV. An 84-trial sequence for one example fMRI run is depicted. Participants completed three fMRI runs

For each participant (*n* = 23), we selected one sweet (SW) and one savory (SV) odor that were matched in rated pleasantness (Fig. 1a)^21,25^. Low intensity (SW_L_, SV_L_) and high intensity (SW_H_, SV_H_) versions of these odors differed in pleasantness (Fig. 1b, repeated measures ANOVA, main effect of intensity: *F*_(1,22)_ = 56.0, *p* = 1.77 × 10^−7^), and there were no differences between either SW_L_ and SV_L_ (paired *t*-test: *t*_(22)_ = 1.16, *p* = 0.26) or SW_H_ and SV_H_ (*t*_(22)_ = 0.18, *p* = 0.86) odor pairs (main effect of identity: *F*_(1,22)_ = 0.35, *p* = 0.56, intensity-by-identity interaction: *F*_(1,22)_ = 0.66, *p* = 0.42). These four odors composed a 2 × 2 factorial design (intensity × identity), and were used as unconditioned stimuli (US) in the subsequent reversal learning task (Fig. 1c). Two randomly selected visual symbols served as conditioned stimuli (CS) for the remainder of the experiment (Fig. 1c).

During the transreinforcer reversal learning task, subjects encountered four unique task states, defined by the pairings between the two CS’s and two of the four US’s (Fig. 1d). Within each state, one CS was paired with a high-intensity US, and the other CS was paired with the low-intensity US of the same odor identity. These deterministic associations were intermittently and covertly changed such that both CS’s were paired with a different US identity, but the same value (identity reversal, iREV), or such that both CS’s were paired with different US values, but the same identity (value reversal, vREV; Fig. 1d). A given state persisted for 9 or 12 trials, and reversals alternated between iREV and vREV throughout the task (Fig. 1f).

On each trial, participants chose one of the two CS’s to receive its currently paired US (Fig. 1e). Two thirds of trials were “forced choice,” wherein participants were cued (and only able) to choose one of the two CS’s. The remaining third were “free choice,” wherein participants could choose either of the two CS’s. Reversals occurred only on forced choice trials, and were followed by another forced choice trial. This experimental design allowed us to examine neural signatures of reward expectation and prediction errors on the forced choice trials, and probe behavior on the free choice trials.

### Choice behavior is sensitive to changes in US value

**Fig. 2:**
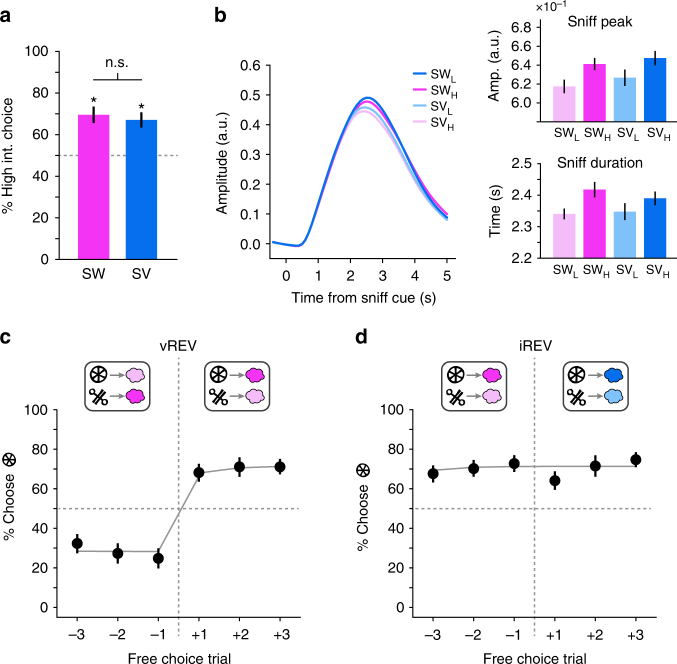
Choice behavior and sniff responses. **a** On free choice trials, choices for the high-intensity version of both the sweet and savory US were above chance and did not differ. Error bars depict across-subjects s.e.m. *p < 0.05, *t*-test vs. chance. **b** Average trial-wise sniff traces, time-locked to sniff cue onset show a main effect of odor intensity on both sniff peak and sniff duration but no effect of odor identity and no interaction. Error bars depicted within-subjects s.e.m. **c** Black circles are the percentage of choices for the CS predicting the high-intensity US (post reversal), plotted for three free choice trials before and after value reversals. Average model-derived choice probabilities are plotted in gray lines. The task state schematics above the plot illustrate an example of one possible value reversal, but the data plotted are averaged across all value reversals. Error bars depict across-subjects s.e.m. **d** Black circles are the percentage of choices for the CS predicting the high-intensity US, plotted for three free choice trials before and after identity reversals. Average model-derived choice probabilities are plotted in gray lines. The task state schematic above the plot illustrates an example of one possible identity reversal, but the data plotted are averaged across all identity reversals and subjects. Error bars depict across-subjects s.e.m

On free choice trials, the CS associated with the high-intensity US was chosen significantly above chance (50%) for both odor identities (SW: *t*_(22)_ = 4.03, *p* = 2.83 × 10^−4^; SV: *t*_(22)_ = 4.20, *p* = 1.83 × 10^−4^; Fig. 2a) and did not differ (*t*_(22)_ = 0.71, *p* = 0.48). Analysis of respiratory activity traces revealed that when cued to sniff the odor outcome on forced choice trials, participants sniffed more for high-intensity odors than for low-intensity odors (repeated measures ANOVA, main effect of intensity on sniff amplitude: *F*_(1,22)_ = 5.95, *p* = 0.023; main effect of intensity on sniff duration: *F*_(1,22)_ = 5.11, *p* = 0.034; Fig. 2b). Sniffing for the two odor identities did not differ (main effect of identity on sniff amplitude: *F*_(1,22)_ = 0.80, *p* = 0.38; identity-by-intensity interaction on sniff amplitude: *F*_(1,22)_ = 0.08, *p* = 0.78; main effect of identity on sniff duration: *F*_(1,22)_ = 0.16, *p* = 0.69; identity-by intensity interaction on sniff duration: *F*_(1,22)_ = 0.93, *p* = 0.35). Participants thus demonstrated an equal preference for the high-intensity versions of both odor identities.

To test whether decisions were sensitive to changes in CS-US associations, we analyzed the proportion of high-intensity choices in free choice trials in a two-way repeated measures ANOVA with reversal type (vREV, iREV) and time as factors (three pre-reversal and three post-reversal trials). We found a significant interaction between reversal type and time (*F*_(5,110)_ 21.30, *p* = 1.02 × 10^−11^), demonstrating that subjects changed their choice behavior with value reversals (one-way ANOVA with trial as a factor containing 6 levels, *F*_(5,110)_ = 27.79, *p* = 3.34 × 10^−10^; Fig. 2c) but not identity reversals (*F*_(5,110)_ = 1.86, *p* = 0.131; Fig. 2d). As expected, this change in behavior on value reversals was also evident in a *post hoc t*-test comparing trials immediately before and after reversals (*t*_(22)_ = 5.98, *p* = 5.14 × 10^−6^). There was also a significant, albeit temporary, decrease in high-value choices after identity reversals (*t*_(22)_ = 2.38, *p* = 0.026), although post-reversal choices remained well above 50% chance (*t*_(22)_ = 3.63, *p* = 0.0015). Interestingly, a similar decrease in optimal value-based choices after identity reversals has previously been observed in rodents^8^. Thus, participants rapidly learned the new CS-US associations after value reversals and adjusted their choices accordingly, but continued to choose the cue paired with the high-intensity US after identity reversals.

### Prediction error signals for identity and value in the midbrain

We next investigated whether violations in reward value and identity expectation evoked prediction error signals in the midbrain. We used a standard reinforcement learning model (see Methods section) to derive trial-by-trial estimates of iPE, positive vPE, and negative vPE (based on prior studies demonstrating that positive and negative value PE’s are processed in dissociable brain regions^30,31^, the vPE trace was split into positive and negative components). As can be seen in Fig. 2c, this model accounted well for choice behavior (average Fisher’s *z*-transformed correlation between model-predicted and actual choice behavior across trials, *z* = 0.48, one-sample *t*-test, *t*_(22)_ = 6.04, *p* = 4.46 × 10^−6^). Model-derived PE’s were included as parametric modulators of finite impulse response functions (FIR) time-locked to the onset of the US’s, and regressed against the fMRI responses in each voxel (see Methods section).

**Fig. 3:**
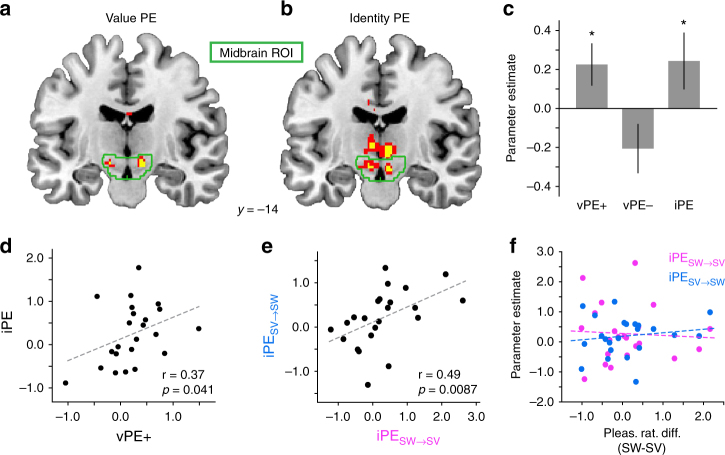
fMRI activity related to iPE and vPE in the midbrain. **a** Positive value prediction errors (PE) evoked fMRI activity in the midbrain. **b** Identity PE evoked fMRI activity in the midbrain. In **a** and **b**, red = *p* < 0.005, uncorrected; yellow = *p* < 0.001, uncorrected. **c** Activity averaged across voxels in the midbrain region of interested (ROI) was significant for both iPE and vPE+. Error bars depict within-subjects s.e.m. *p < 0.05, post hoc* t*-tests vs. zero. **d** Midbrain responses to vPE+ and iPE were correlated across participants. **e** Midbrain responses to the two possible types of identity reversals, iPE_SW→SV_ and iPE_SV→SW_, were correlated across participants. **f** Differences in pleasantness ratings between SW and SV odors were not correlated with midbrain fMRI activity related to iPE_SW→SV_ or iPE_SV→SW_

**Fig. 4:**
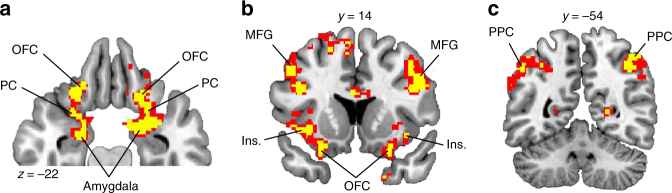
Neural correlates of iPE outside the midbrain. Outside the midbrain ROI, whole-brain analysis of correlations with iPE revealed responses in **a** piriform cortex (PC), orbitofrontal cortex (OFC), amygdala, **b** middle frontal gyrus (MFG), insula (Ins.), and **c** posterior parietal cortex (PPC). T-maps are displayed at *p* < 0.005, uncorrected (red) and *p* < 0.001, uncorrected (yellow). For a complete list of peak coordinates and effect sizes related to this analysis, see Supplementary Table 1

In line with previous results across species^1,3,4,29,32,33^, US-evoked responses in the bilateral midbrain were significantly correlated with positive vPE’s (left, *x* = −12, *y* = −16, *z* = −10, *t*_(22)_ = 4.93, *p*_FWE_ = 0.004; right, *x* = 10, *y* = −14, *z* = −10, *t*_(22)_ = 4.12, *p*_FWE_ = 0.020; Fig. 3a). We found no regions that correlated with negative vPE’s. A separate model with combined positive and negative vPE’s revealed responses in left amygdala (*x* = −22, *y* = −6, *z* = −6, *t*_(22)_ = 4.31) and left ventral striatum (*x* = −20, *y* = 20, *z* = −2, *t*_(22)_ = 4.13). Most importantly, despite the fact that the two food odor identities were matched in pleasantness, we also found significant midbrain responses to violations in expected outcome identity (iPE’s, left, *x* = −6, *y* = −14, *z* = −12, *t*_(22)_ = 3.62, *p*_FWE_ = 0.040; right, *x* = 6, *y* = −14, *z* = −14, *t*_(22)_ = 4.05, *p*_FWE_ = 0.033; Fig. 3b). Additionally, whereas responses to vPE’s were restricted to the midbrain, responses to iPE’s were found in other cortical and subcortical areas, including the OFC, piriform cortex, amygdala, lateral prefrontal cortex, and posterior parietal cortex (Fig. 4 and Supplementary Table 1).

To directly compare PE responses for value and identity, we extracted parameter estimates for both iPE and vPE from an anatomically defined midbrain region of interest (ROI) consisting of the substantia nigra (SN) and ventral tegmental area (VTA)^34^. Interestingly, responses to iPE’s and positive vPE’s (iPE, *t*_(22)_ = 1.69, *p* = 0.050; positive vPE, *t*_(22)_ = 2.12, *p* = 0.022; Fig. 3c) were significantly correlated across subjects (*r* = 0.37, *p* = 0.041; Fig. 3d), suggesting that overlapping neuronal populations in the midbrain may respond to errors in identity and positive value prediction^17^.

Although the SW and SV odor US’s were closely matched in pleasantness for each subject (see Fig. 1b), it remains possible that subtle differences in pleasantness between odors could have elicited value-based prediction errors during identity reversals, potentially confounding iPE with vPE. To rule out this possibility, we conducted a control analysis comparing iPE’s corresponding to switches from SW to SV (iPE_SW→SV_) to iPE’s corresponding to switches from SV to SW (iPE_SV→SW_). If pleasantness differences between SW and SV caused a vPE during identity reversals, these two iPE responses should be negatively correlated. Conversely, if the error signal on identity reversals is computed as an unsigned sensory mismatch, in the absence of a value component, these two separate iPE’s should be positively correlated. In line with the idea that midbrain responses were driven by value-neutral violations in identity prediction, we found a positive correlation between iPE_SW→SV_ and iPE_SV→SW_ (*r* = 0.49, *p* = 0.0087; Fig. 3e). Moreover, there was no correlation between pleasantness differences for the two odors (SW–SV), and midbrain responses to either iPE_SW→SV_ (*r* = −0.06, *p* = 0.79) or iPE_SV→SW_ (*r* = 0.15, *p* = 0.50; Fig. 3f), providing further confirmation that the midbrain iPE signals observed here were not driven by potential value differences between the two odor identities. We conducted several additional control analyses to verify that changes in odor pleasantness as measured throughout the task (which were also included as nuisance regressors in all GLMs) did not account for the observed midbrain effects (Supplementary Fig. 1).

### Encoding of expected outcome identity in the OFC

Analysis of the US-evoked fMRI data revealed that identity reversals elicited identity prediction error signals in the midbrain that co-localized with responses to traditional value PE on value reversals. In theory, these identity-based error signals could be used to update expectations about the identity of future outcomes, which have previously been reported in the OFC^8,25,26^.

**Fig. 5:**
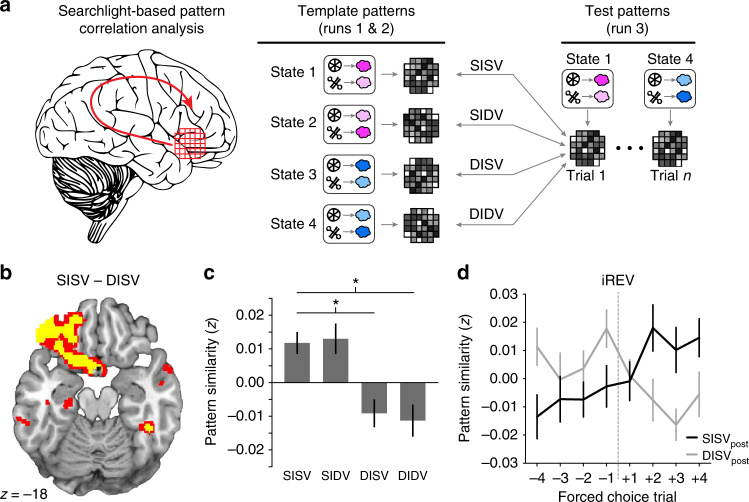
fMRI patterns in OFC encode outcome identity expectations. **a** For each searchlight sphere, “template” patterns from CS-evoked activity of each task state were constructed using fMRI data from two of the three fMRI runs. In the “left out” run, the CS-related activity pattern on each trial was correlated with the four template patterns, and resulting correlation coefficients were labeled according to the relationship to the current state (SISV = same identity, same value; SIDV = same identity, different value; DISV = different identity, same value; DIDV = different identity, different value). Sorted correlation coefficients were Fisher’s *z*-transformed and mapped to the center voxel of the sphere to generate maps of pattern information content for group analysis. **b** Regions in the OFC encoding identity expectations, determined using the SISV–DISV contrast. Red = *p* < 0.005, uncorrected; yellow = *p* < 0.001 uncorrected. **c** Post hoc analysis of pattern similarity at the peak OFC voxel resulting from the SISV–DISV contrast revealed that there was also a significant difference between SISV and DIDV. Error bars depict within-subjects s.e.m. *p < 0.05, paired *t*-tests. **d** For illustration, pattern similarity values at the peak OFC voxel are shown for trials immediately before and after identity reversals (iREV). Correlations are depicted for SISV and DISV templates (relative to their post-reversal relationship) across pre- and post-reversal trials

To probe representations corresponding to expected outcome identity at the time of the CS, we used a multivariate searchlight-based pattern analysis^35–37^. At each searchlight location, and for each forced choice trial, we computed the correlation between the pattern of CS-evoked fMRI activity and four “template” patterns corresponding to the four task states, defined using CS-evoked fMRI responses in independent data (Fig. 5a, see Methods section). These correlations were then sorted according to the relationship between the current trial and the four state templates: same identity, same value (SISV); same identity, different value (SIDV); different identity, same value (DISV); different identity, different value (DIDV, Fig. 5a). Of note, value refers to the specific association between CS’s and the value of the US, not to different levels of expected value (i.e., all states have the same expected value).

We tested for regions that encoded expected outcome identity by comparing states that only differed in identity but not CS-value associations (SISV–DISV). In line with previous work demonstrating expected outcome identity encoding in the OFC^8,21,25,26^, we found significantly higher correlations for SISV compared to DISV in the OFC (Fig. 5b, *x* = −32, *y* = 28, *z* = −14, *t*_(22)_ = 4.35, *p*_FWE_ = 0.0033; see Supplementary Fig. 2 for individual voxel-wise maps of identity signals in this OFC cluster). Post hoc* t*-tests in this region also revealed a difference between states that differed in both expected outcome identity and CS-value associations (SISV–DIDV, *t*_(22)_ = 3.79, *p* = 0.001), but no difference between states with the same expected identity but different CS-value pairing (SISV–SIDV, *t*_(22)_ = 0.24, *p* = 0.81; Fig. 5c). As illustrated in Fig. 5d, expected identity representations in the OFC changed on a trial-by-trial basis following identity reversals.

A parallel comparison between task states that differed only in CS-value but not identity expectations (i.e., SISV–SIDV) revealed that specific CS-value associations were encoded in the amygdala (Supplementary Fig. 3 and Supplementary Fig. 4). These findings underscore prior studies demonstrating that while amygdala and OFC are both critically important for signaling predictive reward information^38–41^, they may do so using different computations^42–44^.

### Midbrain iPE signals update identity expectations in the OFC

**Fig. 6:**
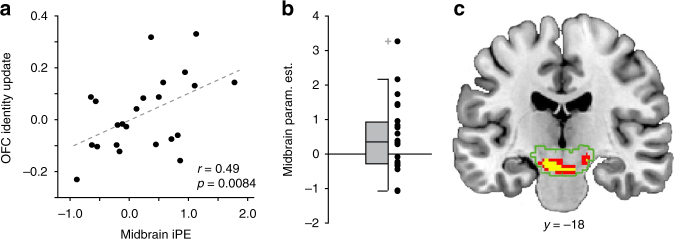
Relationship between midbrain iPE and changes in OFC identity expectations. **a** The magnitude of the iPE response in the midbrain ROI is plotted against the change in pattern-based OFC identity expectations from the trial before to after identity reversals. Each dot represents one subject. **b** Parameter estimates in an anatomical midbrain ROI (green outline in **c**) from a trial-by-trial regression of the change in OFC identity expectations on US-evoked activity. The box plot on the left shows the median, quartiles, data extremes, and outliers. Black dots to the right of the box plot represent individual subject data points. **c** Within the midbrain ROI (green outline) there was a cluster of voxels showing a significant relationship with the change in pattern-based OFC identity expectations. Red = *p* < 0.05, uncorrected, and yellow = *p* < 0.01 uncorrected

Activity patterns in the OFC encoded the identity of the expected outcomes, whereas midbrain activity responded to violations of these identity expectations. Computationally, the central function of prediction errors is to update, or “teach,” associations between predictive events and future outcomes^7^. Given that OFC receives direct projections from midbrain dopamine neurons^45^, we next tested whether midbrain iPE signals are involved in updating identity expectations in the OFC by correlating iPE-related midbrain responses with the degree to which identity expectations changed after identity reversals (“OFC identity update”). Indeed, we found that in the OFC, changes in expected identity from pre to post identity reversals were significantly correlated with the magnitude of midbrain iPE’s (*r* = 0.48, *p* = 0.0097; Fig. 6a). Importantly, neither the OFC identity update nor the midbrain iPE response was correlated with the subject-specific learning rates or the inverse temperature parameters estimated by the RL model (*p* > 0.12), suggesting that this effect is not driven by individual differences in model parameters. Moreover, the correlation between midbrain iPE and OFC identity update remained significant (*r* = 0.46, *p* = 0.026) when iPE’s were derived from a model with the group-average learning rate rather than individual learning rates.

To further test whether midbrain responses correlated with changes in OFC identity expectations on a trial-by-trial basis, we conducted an additional GLM analysis in which changes in pattern-based OFC identity expectations were used as parametric modulators of US-evoked midbrain activity (see Methods section). This analysis probes the degree to which a US-evoked response on trial *t* is related to a change in CS-evoked OFC identity information from trial *t* to trial *t* + 1. At the group level, mean parameter estimates extracted from the midbrain ROI were significantly above zero (*t* = 2.19, *p* = 0.039, one-sample *t*-test, Fig. 6b, c). Post hoc analyses revealed that no other regions associated with iPE signals showed a similar relationship with changes in pattern-based OFC identity expectations (*p* > 0.10, see Supplementary Table 1). Together, these findings provide further evidence that midbrain activity drive updates in identity expectations in the OFC.

## Discussion

Model-based behavior requires neural representations of the sensory and contextual features that constitute a task state, as well as knowledge about the transitions between these states. Although these state representations and transitions play a fundamental role in supporting model-based behavior^11^, the mechanisms by which they are learned and updated have remained elusive. Here we show that one important feature of the task state, the sensory identity of an expected reward, is learned using a midbrain prediction error mechanism akin to traditional learning of cached values^3,46^. Critically, this identity prediction error signal was correlated with trial-by-trial changes in OFC identity expectations, providing a direct link between this learning signal and the information that is updated in a downstream cortical target.

We found overlapping responses to errors in both value and identity expectations in the midbrain. Previous imaging work has shown that the human midbrain responds to value prediction errors^29,32,47^. In addition, human studies have identified state-based prediction error signals in the midbrain^29,48,49^, as well as in the lateral prefrontal cortex and posterior parietal cortex^50,51^. We observed similar widespread responses to identity prediction errors in prefrontal, olfactory, limbic, and posterior parietal regions. Identity prediction errors in lateral prefrontal cortex might be related to this region’s involvement in learning causal relationships between cues and outcomes^50^. Posterior parietal cortex has been linked to sensory evidence accumulation and mismatch^52^, surprise signals have been shown in OFC^29^ and striatum^53^, and risk prediction errors have been observed in insula^54^. The unexpected, value-matched changes in odor identity that subjects experienced in the present study may have involved elements of each of these processes. However, our results suggest that identity-based error signals in midbrain are unique in that only they are directly linked to identity-based updating in downstream OFC.

Our task was designed to independently violate expectations regarding the value and the value-neutral identity of rewarding outcomes, providing direct evidence that both types of errors evoke responses in overlapping regions of the dopamine-rich midbrain. However, given the nature of fMRI, we cannot determine the neurochemical origin of these error signals. The identified area in the midbrain, consisting of VTA and SNc, encompasses a heterogeneous population of dopaminergic, glutamatergic, and GABAergic neurons^55^. In this regard, it is important to note that our results directly parallel recent findings in rodents, which demonstrate that the same dopaminergic neurons respond to errors in the value and identity of expected rewards^17^. Together, these findings support the idea that dopamine plays a much broader role for learning that is not restricted to learning the cached value of rewards.

Our findings show that value-neutral error signals in the midbrain are related to updating of value-neutral associations between predictive events and outcomes. Specifically, our pattern-based analysis revealed signals related to the identity of the expected reward in the OFC^8,25,26^. As in our data, work in rodents has shown that these predictive identity signals in the OFC change rapidly after identity reversals^8^. Critically, our results show that across trials, this change correlates with the strength of midbrain responses, suggesting that midbrain error signals are directly involved in updating value-neutral associations in the OFC.

Unlike several other human imaging studies^51,56–63^, we did not observe a value prediction error in the striatum. This is in line with the suggestion that the striatum responds to value prediction errors only when the timing, but not the magnitude of the predicted reward is violated^47^.

Due to its direct and reciprocal connections with primary olfactory cortex^64^, OFC has been conceptualized as a secondary olfactory cortex^65^. This view is supported by studies across species demonstrating sensory-like odor-evoked responses in OFC, often in the absence of associative cues or perceptual judgments^66–70^. Thus it is possible that OFC has relatively privileged access to olfactory information, and may generate identity-based expectations for odors more readily than for other sensory modalities. Although other studies have shown evidence for identity-based reward representations in the OFC using both olfactory^21,25,26^ and non-olfactory rewards^26,71^, we speculate that olfaction constitutes a uniquely advantageous modality with which to investigate identity-specific encoding in this region.

Taken together, our findings support a role for dopamine in learning the value-neutral associative structure, or model, of the task. This associative structure, consisting of states and the transitions between them, is the basis of model-based control of behavior and has been suggested to be encoded in the OFC^9,22,72^. Thus, in contrast to a relatively narrow role in facilitating model-free learning of cached values, midbrain dopamine signaling may lie at the core of a system supporting predictive coding of any task-relevant features.

## Methods

### Subjects

Twenty seven healthy human participants with no history of psychiatric illness (nine male, ages 19–34, mean ± SD = 25.5 ± 4.1 years) gave informed written consent to participate in this study. The study protocol was approved by the Northwestern University Institutional Review Board. Four participants were excluded from all analyses: two due to excessive head motion during scanning (>4 mm), and two due to a high number of missed responses (>20%). All results presented here are from the 23 remaining participants.

### Odor stimuli and presentation

Eight food odors, including four sweet (strawberry, caramel, cupcake, and gingerbread) and four savory (potato chips, pot roast, sautéed onions, and garlic), were provided by International Flavors and Fragrances (New York, NY). For all experimental tasks, odors were delivered directly to participants’ noses using a custom-built computer-controlled olfactometer capable of redirecting medical grade air with precise timing at a constant flow rate of 3.2 L/min through the headspace of amber bottles containing liquid solutions of the food odors. The olfactometer is equipped with two independent mass flow controllers (Alicat, Tucson, AZ), allowing for dilution of odorants with odorless air. There was a constant stream of odorless air delivered to participants’ noses throughout the experiment, and odorized air was mixed into this airstream at specific time points, without any change in the overall flow rate. Thus, odor presentation did not involve a change in somatosensory stimulation.

### Experimental design

The experiment consisted of two separate days of testing. On both days, participants were instructed to arrive in a hungry state, having fasted for at least 6 h prior to testing. All behavioral ratings were made on visual analog scales using a scroll wheel and mouse button press. Anchors for pleasantness ratings were “most liked sensation imaginable” and “most disliked sensation imaginable.” Anchors for intensity ratings were “strongest imaginable” and “undetectable.” Identity rating anchors corresponded to two-letter abbreviations of the two food odor rewards (e.g., SB for strawberry and PC for potato chip). Subjects were compensated with $20 per h of behavioral testing, and $40 per h of fMRI scanning.

Day 1: Stimulus selection and task familiarization: Participants first provided pleasantness ratings of the eight food odors. One sweet odor and one savory odor were chosen based on these ratings such that they were closely matched in pleasantness. Next, we acquired pleasantness ratings for the two selected odors across a range of odor concentrations, diluted to varying degrees with odorless air. Based on these ratings, we selected two intensity levels for each odor, such that the two low-intensity odors had the same pleasantness and the two high-intensity odors had the same pleasantness. Next, participants provided independent pleasantness and intensity ratings on these four odors to verify the relationship between intensity and pleasantness (i.e., value).

Participants next completed 84 consecutive trials of the transreinforcer reversal learning task they would eventually complete in the fMRI scanner on Day 2. For this task, two abstract visual symbols were randomly chosen to serve as conditioned stimuli (CS) throughout the rest of the experiment. Each trial started with a trial type “cue” phase in which either one of the two CS’s (indicating it was a forced choice trial) or a question mark (indicating it was a free choice trial) appeared for 4 s. In the subsequent “choice phase,” both CS’s were presented in white font on either side of a white crosshair (side fully randomized and counterbalanced). Participants had 1.5 s to choose via left or right mouse click the CS that appeared in the preceding “cue” phase (in the case of a forced choice trial), or whichever CS they preferred (in the case of a free choice trial). If no response was made within 1.5 s of choice onset, “TOO SLOW” appeared on the screen and the next trial was initiated after a variable delay. If a response was made, the unselected CS turned gray, and the odor (unconditioned stimulus, US) currently paired with the selected CS was delivered 2 s later. Odor delivery was indicated by changing the color of the center crosshair to blue, informing participants to sniff to receive the US. After this 3 s “outcome” phase, participants rated (max duration 3.5 s) either the pleasantness or identity of the odor (rating type randomized), followed by a 0–2 s inter-trial interval. Across the 84 trials, the choice task was covertly subdivided into 8 blocks of trials delineated by the specific CS–US associations predetermined for that block. Each block consisted of either 9 or 12 trials, and the length of blocks across the session was pseudorandomized. Within a given block, one of the CS’s was paired deterministically with the high-intensity version of one odor identity (e.g., sweet high: SW_H_), while the other CS was paired deterministically with the low-intensity version of the same odor identity (e.g., sweet low: SW_L_). After each block, the CS–US associations were changed without warning, and new blocks always began with two forced choice trials (one for each CS). In the case of reward identity reversals, the expected identity of the US was changed for both CS’s while leaving CS-value associations the same. In the case of reward value reversals, the CS-value association was swapped between the two CS’s, while leaving expected identity the same. Reversals alternated between identity and value, and there were seven total reversals across the 84-trial task.

Day 2: Transreinforcer reversal learning task and fMRI scanning: The Day 2 fMRI scanning session, conducted within ~10 days (mean ± SD = 10.0 ± 4.4 days) of the Day 1 behavioral session, started with a brief reminder of the four odor US’s chosen on Day 1, followed by odor pleasantness ratings. Participants then completed three runs (84 trials each) of the reversal learning task described above while undergoing fMRI scanning. Each run lasted ~21 min, and the sequence of alternating identity and value reversals was counterbalanced across subjects.

### fMRI data acquisition

MRI data were acquired on a Siemens 3T PRISMA system equipped with a 64-channel head-neck coil. Echo-Planar Imaging (EPI) volumes were acquired with a parallel imaging sequence with the following parameters: repetition time, 2 s; echo time, 22 ms; flip angle, 90°; multi-band acceleration factor, 2; slice thickness, 2 mm; no gap; number of slices, 58; interleaved slice acquisition order; matrix size, 104 × 96 voxels; field of view 208 mm × 192 mm. The functional scanning window was tilted ~30° from axial to minimize susceptibility artifacts in OFC^73^. Each fMRI run consisted of 640 EPI volumes covering all but the dorsal portion of the parietal lobes. To aid in co-registration and normalization of the functional scans, we also acquired ten EPI volumes for each participant covering the entire brain, with the same parameters as described above except 95 slices and a repetition time of 5.25 s. A 1 mm isotropic T1-weighted structural scan was also acquired for each participant. This image was used for spatial normalization.

### Sniff recording and analysis

During scanning, breathing activity was monitored using a respiratory effort band (BIOPAC Systems Inc, Goleta, CA) affixed around the participant’s torso, and recorded using PowerLab equipment (ADInstruments, Dunedin, New Zealand) at a sampling rate of 1 kHz. Breathing traces for each fMRI run were smoothed using a moving window of 250 ms, high-pass filtered (cutoff, 50 s) to remove slow-frequency drifts, normalized by subtracting the mean and dividing by the standard deviation across the run trace, and down-sampled to 0.5 Hz for use as nuisance regressors in fMRI data analyses (see below).

For analysis of sniff peak amplitude and sniff duration, trial-specific sniff traces were baseline corrected by subtracting the mean signal across the 0.5 s window preceding sniff cue onset, and then normalized by dividing by the maximum sniff amplitude of all trials in the run. Sniff amplitude was then calculated as the max signal within 5 s of sniff cue onset, and sniff duration was calculated as the time from sniff cue onset to max amplitude. Trial-by-trial measures of sniff peak amplitude and duration were sorted by condition and tested at the group level for significant effects.

### Reinforcement learning model

To generate trial-by-trial estimates for prediction errors, we implemented a standard reinforcement learning (RL) model^6,7^. This model independently learned and updated identity and value expectations via prediction errors for identity (iPE) and value (vPE), respectively

$${\mathrm{iPE}}_t = I_t - {\mathrm{EI}}_t$$

$${\rm vPE}_t = V_t - {\mathrm{EV}}_{S,t}$$

Here EI and EV indicate expected identity and expected value, respectively*. *EV was initialized at 0 for each CS. *V* is the value (i.e., intensity) of the odor delivered on trial *t* and was set to 0 and 1 for low- and high-intensity odors, respectively. EI was initialized at 0.5. *I* is the identity of the delivered odor and was set to 0 and 1 for savory and sweet odors, respectively. EV for the selected CS (EV_*S*_) was updated using a learning rate (*α*) according to

$$\mathrm{EV}_{S,t + 1} = \mathrm{EV}_{S,t} + \alpha \ast \mathrm{vPE}_t$$

Within a given block of trials in our task, the value of the outcomes associated with the two CS’s was anti-correlated. We therefore incorporated a fictive (i.e., counterfactual) prediction error (fPE) to update the EV of the non-selected CS (EV_NS_), as in previous studies with similar designs^74–76^

$${\mathrm{fPE}}_t = \left( { - V_t + 1} \right) - {\mathrm{EV}}_{{\mathrm{NS}},t}$$

$${\mathrm{EV}}_{{\mathrm{NS}},t + 1} = {\mathrm{EV}}_{{\mathrm{NS}},t} + \alpha \ast {\mathrm{fPE}_{\mathrm{t}}}$$

Note that the term (−*V*_*t*_ + 1) inverts the value of the delivered odor, and thus reflects the value of the odor that would have been obtained if the other CS had been selected. Within a given block of trials in our task, the identity of the outcomes paired with the two CS’s was the same. We therefore included a single EI term in the model, and assumed that EI was updated simultaneously for both CS’s according to

$$\mathrm{EI}_{t + 1} = \mathrm{EI}_t + \alpha \ast \mathrm{iPE}_t$$

The model used the EV for the two CS (EV_1_ and EV_2_) to generate trial-wise choice probability for the two CS [*P*_1,*t*_ and *P*_2,*t*_ = (1 − *P*_1,*t*_)] according to a softmax rule with slope *θ* (*θ* = 3^c^ − 1; parameterizing the slope in this way accounts for deterministic choice strategies that tend to be adopted in binary decision tasks^77–79^)

$$P_{1,t} = \frac{{{\rm e}^{\theta \ast {\rm EV}_{1,t}}}}{{{\rm e}^{\theta \ast {\rm EV}_{1,t}} + {\rm e}^{\theta \ast {\rm EV}_{2,t}}}}$$

The two free parameters of the model (*α* and *c*) were estimated for each subject using Bayesian hierarchical parameter estimation, based on previous work^78^. In brief, parameters of individual participants are assumed to be generated from parent distributions that are modeled as independent beta distributions with parameters for means and standard deviations taken from normal and uniform distributions, respectively. We used choice data from all participants to compute the posterior distributions for the two parameters. Posterior inference was performed using the Markov Chain Monte Carlo (MCMC) sampling scheme as implemented in the Stan software package for Matlab (MatlabStan, mc-stan.org/users/interfaces/matlab-stan). A total of 3000 samples were drawn after 1000 burn-in samples with three chains.

Our model used the same learning rate to update EI based on iPE, and to update EV based on vPE and fPE. We tested alternative models with independent learning rates for vPE and fPE. Models were compared using the Akaike Information Criterion (AIC) and Bayesian Information Criterion (BIC). The model with a single learning rate outperformed models with independent learning rates for vPE and fPE (AIC = 2223.59 vs. 2270.67; BIC 2334.32 vs. 2436.78), and was therefore used for analysis of fMRI data. We also compared this model with a model without a fictive PE component. The model without the fictive PE performed slightly worse that the model including the fPE (AIC = 2224.12, BIC = 2334.86).

### fMRI data preprocessing

All image preprocessing and general linear modeling was done using SPM12 software (www.fil.ion.ucl.ac.uk/spm/). To correct for head motion during scanning, for each subject all functional EPI images across the 3 fMRI runs were aligned to the first acquired image. For the multivoxel pattern correlation analysis (see below), the motion-corrected images were smoothed with a Gaussian kernel at native scan resolution (2 × 2 × 2 mm) to reduce noise but retain potential information content^80^. The ten whole-brain EPI volumes were independently motion corrected and averaged. Both the EPI time series (using the average EPI) and the T1 structural image were then co-registered to the mean whole-brain EPI. For spatial normalization of images to a standardized template, the structural image was normalized to the Montreal Neurological Institute (MNI) space using the 6-tissue probability map provided by SPM12. The deformation fields resulting from this normalization step were applied to brain maps of correlation values. For univariate analyses, the motion-corrected and co-registered EPI images were normalized to MNI space using the previously calculated deformation fields. Both the normalized pattern correlation maps (see below) and the normalized functional EPI volumes were spatially smoothed with a 6 × 6 × 6 mm Gaussian kernel for group-level statistical testing.

### Univariate test for prediction error-related fMRI signals

In order to test for neural responses related to both value (vPE) and identity prediction errors (iPE), we constructed subject-level event-related GLM’s using finite impulse response (FIR) functions specified over nine time bins time locked to the onset of odor US. Each time bin included three parametric modulators, corresponding to the unsigned iPE, positive vPE, and negative vPE estimates derived from the best-fitting reinforcement learning model (with individually estimated parameters) described above. Serial orthogonalization of modulator regressors was turned off for this and all other SPM GLM analyses. Nuisance regressors included: the smoothed, normalized sniff trace, down-sampled to scanner temporal resolution (0.5 Hz); pleasantness ratings acquired during the task; the six realignment parameters (three translations, three rotations) calculated for each volume during motion-correction; the derivate, square, and the square of the derivative of each realignment regressor; the absolute signal difference between even and odd slices, and the variace across slices, in each functional volume (to account for fMRI signal fluctuation caused by within-scan head motion); additional regressors as needed to model out individual volumes in which particularly strong head motion occurred. To account for typical hemodynamic lag (4–6 s) and time to peak inhalation (~2 s) relative to odor cue onset, we constructed subject-level contrast images composed of the average of time bins 3, 4, and 5 for each parametric modulator (corresponding to 6, 8, and 10 s after sniff cue onset). These subject-level contrasts were then tested at the group level using one-sample *t*-tests for significance.

### MVPA for identity expectations and CS-value associations

We implemented a searchlight-based correlation analysis to test for information about specific task states in fMRI activity patterns without potential bias due to voxel selection. Searchlight spheres consisted only of gray matter voxels specified by inclusively masking functional volumes with the gray matter probability map provided by SPM, thresholded at 0.1 and inverse normalized to native space. We considered a task state to be defined by the combined associations between the two CS’s and two US’s assigned to a given block of choice task trials. Thus there were four unique task states in this experiment (see Fig. 1d), each of which occurred twice in each fMRI run. To estimate CS-evoked voxel activity patterns corresponding to these task states, which we refer to as “templates” for later testing, we first specified separate general linear models (GLMs) for each fMRI run using the motion-corrected and smoothed (2 × 2 × 2 mm) functional EPI volumes. These GLMs included two event-related regressors of interest for the forced choice trials of each task state (corresponding to the two occurrences of each state), modeled from the onset of the pre-choice trial type cue to the time a choice was made. These GLMs also included two similar regressors for each task state corresponding to the free choice trials, one event-related regressor time-locked to the onset of all US’s, and one event-related regressor time-locked to the onset of all ratings. Nuisance regressors were the same as those described above for the univariate GLM’s.

We next specified a second set of “test” GLMs for each fMRI run, wherein each trial was modeled as a separate event-related regressor spanning from pre-choice trial type cue to the time a choice was submitted. These “test” GLMs also included regressors for US and rating onset, and the same nuisance regressors that were included in the template GLMs described above. We then used the parameter estimates from these template and test GLMs in a searchlight approach to identify brain regions that encoded specific types of information about task states on a trial-by-trial basis. Within a given searchlight sphere (radius of ~3 voxels) we extracted patterns of parameter estimates from two of the three template GLMs (e.g., runs 1 and 2) corresponding to forced choice regressors specified for each task state, and averaged across runs to generate one template pattern for each of the four task states. Template patterns were decorrelated by subtracting the mean across the four states from each voxel. We then extracted patterns of parameter estimates from the test GLM of the “left out” run (e.g., run 3) corresponding to each single-trial regressor. We then calculated the Pearson correlation (*r*) between each test pattern and each of the four template patterns. This process was repeated three times, with each of the three runs serving as test and the other two runs serving as templates.

The resulting *r* values were Fisher’s *z* transformed ($z = \frac{{{\rm ln}\left( {1 + r} \right) - {\rm ln}\left( {1 - r} \right)}}{2}$) to allow for averaging and statistical testing, and then sorted according to the relationship between the actual state of each trial-specific test pattern and the states of the four template patterns. Correlations between the same states were labeled SISV, and served as a control condition with which to contrast other correlations. Correlations with state templates that differed in expected outcome identity, but not CS-value associations, were labeled DISV, those that differed in CS-value associations, but not expected outcome identity, were labeled SIDV, and those that differed in both features were labeled DIDV. Average (across trials) correlation values corresponding to each of these labels were mapped back to the center voxel of each searchlight sphere, resulting in a unique brain map for each label and subject. Contrasts between conditions (e.g., SISV–DISV) were tested for significance at the group level using paired *t*-tests.

### Relating midbrain activity to OFC identity expectations

For the identity-based analysis, we first created an ROI consisting of a sphere of 3-voxel radius surrounding the peak coordinate of identity-coding OFC. Within this sphere, and for each forced choice trial, we calculated the correlation between that trial’s CS-evoked activity pattern in one fMRI run and the state-specific template patterns from the other two runs. We then computed the difference between the correlations corresponding to the two sweet identity states and savory identity states, resulting in a trial-by-trial measure of identity-based information content. We then computed the absolute difference between identity information content on each trial and the next trial, thus deriving a measure of the trial-by-trial changes in CS-evoked pattern-based identity expectations in OFC. These traces were then used as parametric modulators of US-evoked fMRI activity on forced choice trials in subject-wise GLM’s with FIR as basis functions. Free choices were modeled in a separate regressor, and nuisance regressors were the same as those described above. Subject-wise parameter estimates corresponding to the parametric modulator term (averaged across time bins 3, 4, and 5) were extracted from an anatomical midbrain ROI (and in subsequent post hoc analyses from spheres [3-voxel radius] surrounding other coordinates showing a significant response to iPE’s outside the midbrain), averaged across voxels within this ROI, and subjected to a group-level one-sample *t*-test.

For the analysis testing for a relationship between midbrain activity and updating of CS-value associations (see Supplementary Fig. 4), we first created an ROI consisting of a sphere of 3-voxel radius surrounding the peak coordinate of CS-value coding amygdala. Within this sphere, and for each trial, we calculated the difference in pattern correlation between states that differed in specific CS-value associations (regardless of identity), and then proceeded as described above to implement the change in this correlation difference as a parametric modulator of US-evoked fMRI activity.

### Group-level statistical analysis

For both the univariate and multivariate whole-brain group-level analyses we used voxel-wise *t*-tests. Significance threshold was set at *p* < 0.05, small-volume corrected for multiple comparisons at the voxel level (family-wise error rate, FWE) using anatomical regions of interest in the midbrain^34^, the OFC and amygdala (defined using the Automatic Anatomical Labeling [AAL] atlas).

### Data availability

All relevant data and Matlab codes are available from the authors upon request.

## Electronic supplementary material

Supplementary Information
