# Supplementary material for: Identity prediction errors in the human midbrain update reward-identity expectations in the orbitofrontal cortex
Source: Nat Commun. 2018 Apr 23;9:1611. doi: 10.1038/s41467-018-04055-5 (PMC5913228; doi:10.1038/s41467-018-04055-5)
Supplement: Supplementary file 1 — Supplementary Information [file 41467_2018_4055_MOESM1_ESM.pdf]

SUPPLEMENTARY INFORMATION

Identity prediction errors in the human midbrain update reward-identity expectations in the orbitofrontal cortex

Howard & Kahnt

SUPPLEMENTARY FIGURES

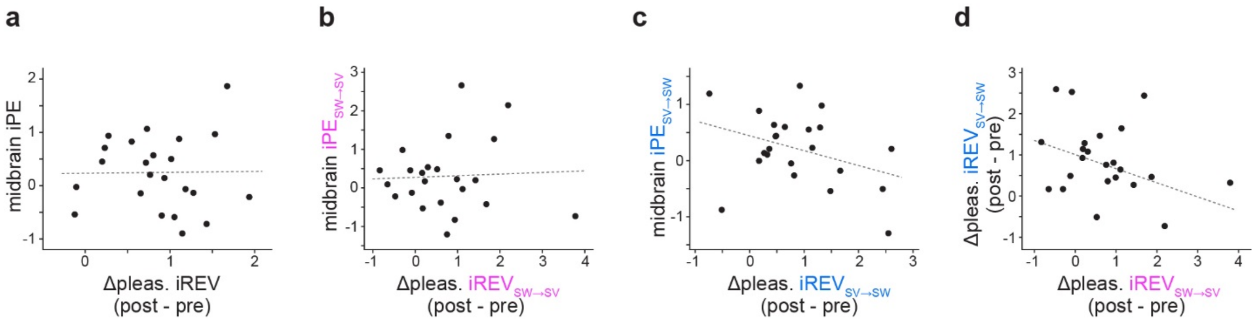

Supplementary Figure 1. Relationship between online pleasantness ratings and midbrain iPE signals. Odor pleasantness ratings were made after outcome delivery on half of the trials (randomly ordered) throughout the task. To test whether there were changes in these ratings after reversals, we compared the first post-reversal ratings made for the outcome that was experienced on a reversal trial to the most recent pleasantness rating made for that same outcome prior to the reversal. These pre and post ratings were then sorted into value (separated by Low→High and High→Low value reversals) and identity reversals. As expected, we found a significant increase in rated pleasantness for the Low→High value reversals ($t_{(22)}$ = 4.34, p = $2.63\text{x}10^{-4}$) and a significant decrease in pleasantness on High→Low reversals ($t_{(22)}$ = -5.70, p = $9.75\text{x}10^{-6}$). We also observed an increase in pleasantness after identity reversals (SW→SV: $t_{(22)}$ = 3.26, p = 0.0036, SV→SW: $t_{(22)}$ = 4.82, p = $8.04\text{x}10^{-5}$). **(a)** However, the average increase in pleasantness on identity reversals was not correlated with the midbrain iPE response (r = 0.014, p = 0.95). **(b)** There was also no correlation between changes in pleasantness on SW→SV identity reversals and corresponding SW→SV midbrain iPE's (r = 0.05, p = 0.83), and **(c)** no correlation between changes in pleasantness on SV→SW identity reversals and corresponding SV→SW midbrain iPE's (r = -0.36, p = 0.09). Thus it is unlikely that changes in perceived pleasantness, as measured throughout the task, explain our observed midbrain iPE effects. **(d)** This point is underscored by the fact that SW→SV pleasantness change and SV→SW pleasantness change themselves are not correlated (r = -0.29, p = 0.19), and thus cannot account for the observed positive correlation between midbrain iPE$_{SW\rightarrow SV}$ and iPE$_{SV\rightarrow SW}$ (see **Fig. 3e**).

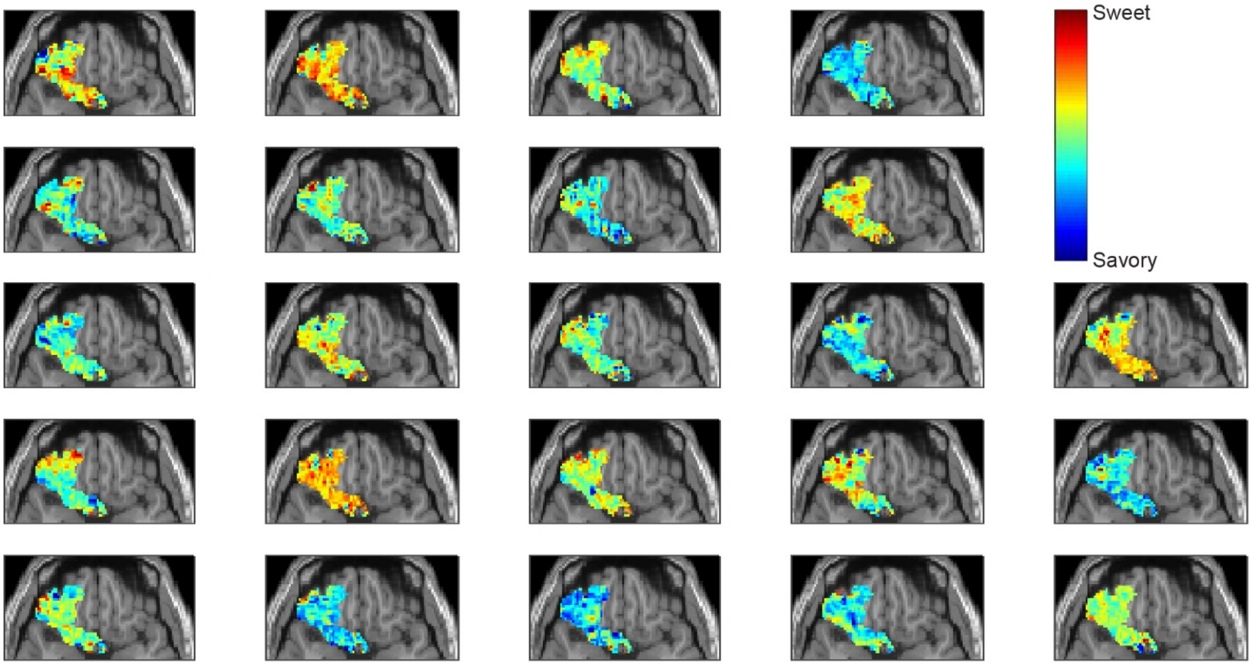

Supplementary Figure 2. Individual identity-based activity patterns in the OFC. In order to visualize the activity patterns driving the observed pattern-based identity information in OFC, we computed individual maps of CS-evoked parameter estimates corresponding to each task state. We then computed the difference between sweet task states (i.e. trial blocks in which both odor outcomes were the sweet odor) and savory task states. Difference maps are shown here on a single axial slice (z = -18) inclusively masked by the region of OFC that significantly (p < 0.005, uncorrected) encodes identity expectations at the group level (see **Fig. 4b**). Each difference map is individually scaled according to the maximum and minimum value within the mask for that subject. Warm colors denote greater activity for expectations of sweet odors, and cool colors denote greater activity for expectations of savory odors (see color bar top right).

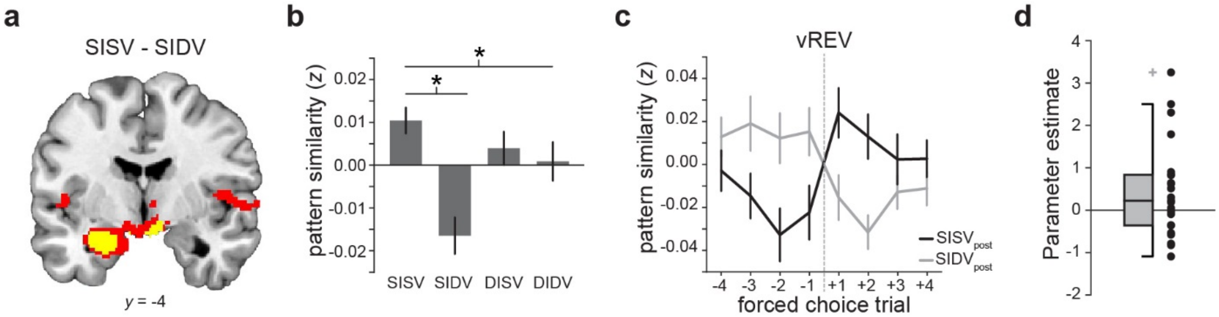

Supplementary Figure 3. fMRI patterns in amygdala encode CS-value associations. (**a**) Voxels in the amygdala (x = -26, y = -4, z = -22, $t_{(22)}$ = 4.54, p_{FWE} = 0.00067) encoding CS-value associations, identified using the SISV-SIDV contrast. Note that "CS-value" refers to the specific association between the two CS's and the value of the associated US's and is not related to differences in overall expected value (SISV and SIDV had the same expected value). Red = p < 0.005, uncorrected, and yellow = p < 0.001, uncorrected. (**b**) *Post hoc* analysis at the peak amygdala voxel resulting from the SISV-SIDV contrast revealed that there was also a significant difference between states with different CS-value and different expected outcome identity (SISV – DIDV, $t_{(22)}$ = 1.86, p = 0.038), but no difference between states with same CS-value but different expected outcome identity (SISV and DISV, $t_{(22)}$ = 1.12, p = 0.28). Error bars depict within-subjects s.e.m. *p < 0.05, *post hoc* paired t-tests. (**c**) For illustration, pattern correlation with SISV and SIDV templates (defined relative to the post-reversal relationships)at the peak amygdala voxel for forced choice trials before and after value reversals (vREV). **(d)** The change in pattern-based information for CS-value associations in a sphere surrounding the peak CS-value coding amygdala coordinate was significantly correlated with trial-by-trial US-evoked midbrain activity ($t_{(22)}$ = 1.92, p = 0.034, one-tailed t-test on parameter estimates corresponding to amgydala pattern change parametric modulator regressor). These findings suggest that midbrain activity updates CS-value associations in the amygdala.

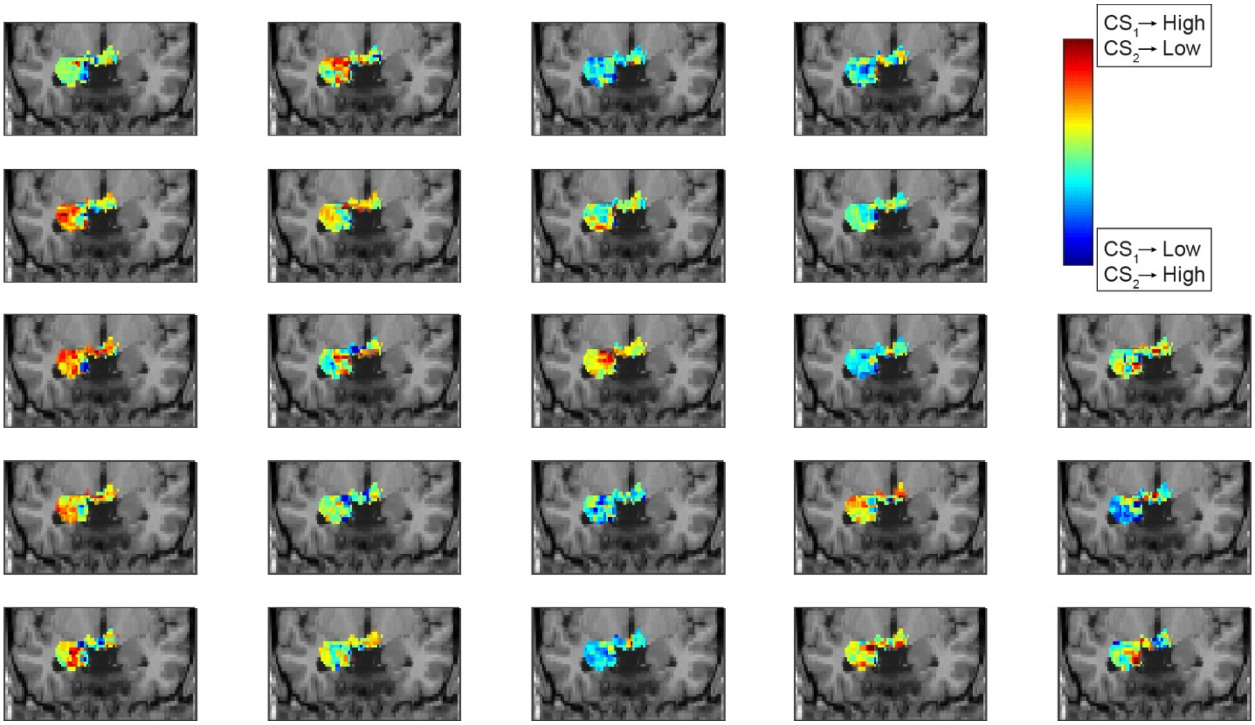

Supplementary Figure 4. Individual CS-value-based activity patterns in the amygdala. We computed individual maps of CS-evoked parameter estimates corresponding to each task state. We then computed the difference between CS_1→High + CS_2→Low task states (regardless of identity) and CS_1→Low + CS_2→High task states. Difference maps are shown here on a single coronal slice (y = -4) inclusively masked by the region of amygdala that significantly ($p < 0.005$, uncorrected) encodes CS-value expectations at the group level (see **Supplementary Fig. 4a**). Each difference map is individually scaled according to the maximum and minimum value within the mask for that subject. Warm colors denote greater activity for expectations of CS_1→High + CS_2→Low task states, and cool colors denote greater activity for expectations of CS_1→Low + CS_2→High task states (see color bar top right).

SUPPLEMENTARY TABLES

Supplementary Table 1. Brain regions outside the midbrain correlating with iPE (*p* < 0.001, uncorrected), and their relation to OFC identity update within- and across-subjects.

Region name	MNI coordinate				Across-subject corr. w/ mean OFC update		Within-subject trial-wise OFC pattern change	
	X	*Y*	*Z*	*t* value	*r*	*P*	*t*	*p*
Right anterior OFC	34	50	-16	5.14	0.18	0.42	0.91	0.38
Left anterior OFC	-36	50	-12	4.97	0.09	0.69	0.18	0.86
Right lateral OFC	46	42	-8	5.55	0.09	0.67	-0.45	0.65
Left lateral OFC	-38	48	-8	4.02	0.15	0.50	-0.32	0.75
Right central OFC	24	34	-18	4.72	0.46	**0.03**	1.14	0.27
Left central OFC	-26	28	-18	5.37	0.21	0.35	1.38	0.18
Right posterior OFC	20	24	-20	6.28	0.14	0.54	0.98	0.34
Left posterior OFC	-26	18	-16	5.37	0.17	0.43	-0.41	0.68
Right piriform cortex	28	4	-20	4.70	0.18	0.42	0.69	0.50
Right amygdala	16	-6	-14	4.43	0.46	**0.03**	-1.10	0.28
Left amygdala	-20	-4	-20	5.00	0.27	0.21	-0.35	0.73
Right middle temporal gyrus	60	-46	-10	6.00	0.09	0.67	0.36	0.72
Right posterior parietal area	46	-56	52	6.15	0.05	0.81	-1.03	0.31
Left posterior parietal area	-50	-58	44	4.89	0.36	0.09	-2.47	0.02
Posterior cingulate cortex	-2	-28	30	6.49	0.26	0.22	1.16	0.26
Right ventral striatum	10	8	-2	4.87	0.27	0.22	0.08	0.93
Right middle frontal gyrus	52	18	32	5.09	0.25	0.25	-0.54	0.60
Left middle frontal gyrus	-40	14	26	4.92	0.06	0.78	0.12	0.90
Right lateral prefrontal cortex	46	38	14	4.29	0.23	0.29	0.21	0.84
Left lateral prefrontal cortex	-46	34	14	5.85	0.19	0.37	-0.24	0.81
Right insula	38	22	-4	5.01	0.11	0.63	-1.12	0.27
Left Insula	-34	16	-6	5.23	0.22	0.31	-1.03	0.32
